# Neuromodulation of metabolic functions: from pharmaceuticals to bioelectronics to biocircuits

**DOI:** 10.1186/s13036-019-0194-z

**Published:** 2019-08-01

**Authors:** Benjamin J. Seicol, Sebastian Bejarano, Nicholas Behnke, Liang Guo

**Affiliations:** 1 Neuroscience Graduate Program, The Ohio State University, Columbus, OH USA; 2 Department of Neuroscience, The Ohio State University, Columbus, OH USA; 3 Department of Food, Agricultural, and Biological Engineering, The Ohio State University, Columbus, OH USA; 4 Department of Electrical and Computer Engineering, The Ohio State University, Columbus, OH USA

**Keywords:** Neuromodulation, Metabolism, Inflammation, Bioelectronic medicine, Biocircuits

## Abstract

Neuromodulation of central and peripheral neural circuitry brings together neurobiologists and neural engineers to develop advanced neural interfaces to decode and recapitulate the information encoded in the nervous system. Dysfunctional neuronal networks contribute not only to the pathophysiology of neurological diseases, but also to numerous metabolic disorders. Many regions of the central nervous system (CNS), especially within the hypothalamus, regulate metabolism. Recent evidence has linked obesity and diabetes to hyperactive or dysregulated autonomic nervous system (ANS) activity. Neural regulation of metabolic functions provides access to control pathology through neuromodulation. Metabolism is defined as cellular events that involve catabolic and/or anabolic processes, including control of systemic metabolic functions, as well as cellular signaling pathways, such as cytokine release by immune cells. Therefore, neuromodulation to control metabolic functions can be used to target metabolic diseases, such as diabetes and chronic inflammatory diseases. Better understanding of neurometabolic circuitry will allow for targeted stimulation to modulate metabolic functions. Within the broad category of metabolic functions, cellular signaling, including the production and release of cytokines and other immunological processes, is regulated by both the CNS and ANS. Neural innervations of metabolic (e.g. pancreas) and immunologic (e.g. spleen) organs have been understood for over a century, however, it is only now becoming possible to decode the neuronal information to enable exogenous controls of these systems. Future interventions taking advantage of this progress will enable scientists, engineering and medical doctors to more effectively treat metabolic diseases.

## Background

Historically treated through pharmaceutical interventions, metabolic functions play a crucial role in the pathophysiology of numerous diseases. Despite the widespread success of pharmacological approaches in treating disease, many problems remain and prevent the alleviation of the symptoms for patients with chronic metabolic illnesses. Sides effects, drug resistance and patient compliance are just a few of these obstacles. Many chronic diseases are, or become, treatment resistant, further limiting the application of pharmaceutical treatments. This has led to a new wave of interest in alternative therapeutic strategies to treat chronic metabolic diseases. A promising approach involves the stimulation of nerves that contribute to the pathology through dysregulation of metabolic functions. Silencing or activating nerves to control organ and tissue functions is referred to as bioelectronic medicine. Rather than pharmaceutical, this approach uses electroceutical interventions to restore function and ameliorate symptoms of disease. Electrical stimulation of the brain and nerves can improve the quality of life in patients suffering from otherwise refractory diseases. However, many challenges remain in the integration of abiotic implants into biological tissues, including foreign body reactions, artificial stimuli and long-term maintenance that requires follow-up invasive surgeries. Strategies using miniaturization, soft materials and biomimicry improve outcomes and prolong device fidelity, however, fundamental limits remain to be overcome. In the case of progressive degenerative diseases, such as Type 1 diabetes (T1D), loss of function due to cell death cannot be replaced through bioelectronic interventions. Engineering rationally-designed multicellular biological circuits, or biocircuits for short, provides a promising solution to overcome the remaining challenges. Autologous, living tissue implants could restore lost tissues and functions, as well as providing life-long, seamlessly biointegrated implants for the treatment of chronic diseases.

## Introduction

**Fig. 1:**
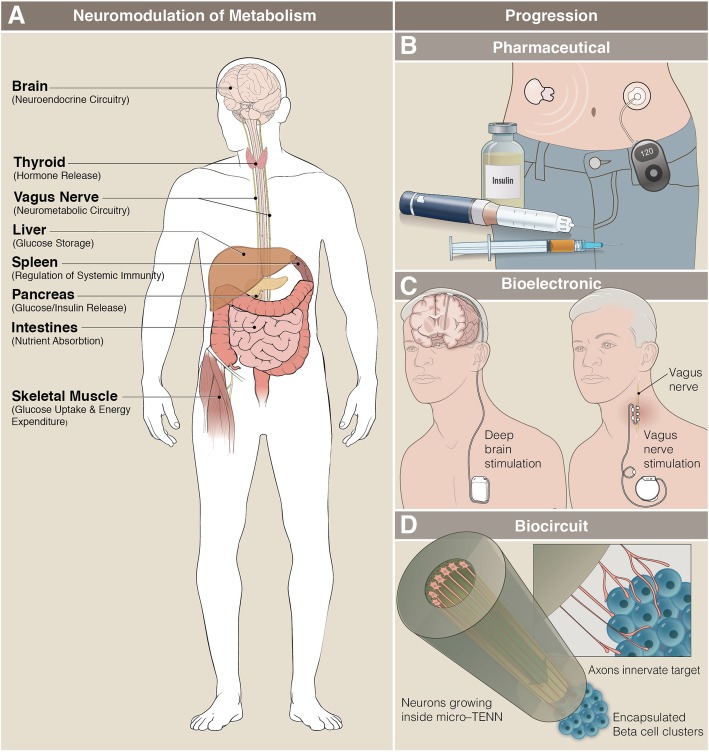
Target organs and progression of neuromodulation technologies to control metabolic functions. Neuromodulation can be categorized based on the peripheral target innervated by the circuit or nerve stimulated. **a**. Target organs that regulate metabolism are innervated by afferent and efferent fibers that release neurotransmitters or paracrine signals which modulate the organ’s function and greatly impact local and systemic metabolisms. **b**. Pharmaceutical interventions for T1D. Blood glucose level is self-measured, and insulin is injected via syringe multiple times daily. Closed-loop advanced drug delivery systems greatly improve disease management outcomes and patients’ life quality. **c**. DBS and VNS systems for bioelectronic medicine require implanted stimulators that generate electrical pulses. They are then connected by wires to microelectrodes implanted in the brain or on the vagus nerve. **d**. Using a hydrogel-based micro-TENN as scaffold [53], neuronal networks can be rationally designed and transplanted to innervate and/or replace living tissues. An autologous β-cell biocircuit concept consists of ACh releasing neurons inside a micro-TENN with directed innervation into vascularized, mature and encapsulated β-cell clusters derived from patient’s iPSCs. Image courtesy of Anthony S. Baker and Courtney Fleming, The Ohio State University^©^ 2019; produced with permission.

Neuromodulation of metabolic functions is an exciting approach for restoring health through targeted stimulation of neural circuitry innervating organs and tissues. Metabolism is defined as cellular events that involve catabolic and/or anabolic processes, including control of systemic metabolic functions, as well as cellular signaling pathways, such as cytokine release by immune cells. Compared with neuromodulation of behaviors, electrical stimulation to modulate metabolic functions results in subtler, but no less important, changes in physiology (see Fig. 1a). Electrical stimulation can restore dysfunctional neurometabolic circuitry [36, 37, 74] and may provide a new therapeutic avenue for metabolic diseases. Central and peripheral neurometabolic circuitry can be stimulated to modulate both systemic and local metabolisms [7]. As such, bioelectronic medicine promises to provide relief for patients suffering from refractory metabolic conditions [3, 21, 48, 71].

Metabolic functions extend beyond processes that control systemic metabolism. All cellular signaling pathways, for instance the production and release of cytokines by resident immune cells, also belong to metabolic functions subject to regulation by neuronal circuits. Cytokines are protein signals produced and secreted primarily by immune cells that trigger changes in immune function, such as inflammation. Inflammation is characterized by swelling, redness, heat and pain and is driven by an increased production and release of pro-inflammatory cytokines typically from resident immune cells (e.g. macrophages). Neurogenic inflammation — neural regulation of immune responses — was first discovered over 100 years ago [10]. Sensory nerves regulate immune function, and when stimulated, can reduce local inflammation and immune responses [27, 28]. Autonomic nerves innervate primary and secondary lymphoid organs, such as bone marrow and spleen, respectively [44]. Neural-immune interactions allow for dynamic regulations of both systemic and local inflammations through neuroimmune circuits [105]. Understanding neural regulation of metabolic functions, including glycemic control and immunity, can allow unprecedented access to treat diseases underserved by pharmaceutical therapeutics.

Historically treated through pharmacological therapies, metabolic disorders, such as T1D, are now routinely treated through advanced technology-assisted pharmaceutical interventions that employ biosensors [80] and closed-loop drug delivery systems [4, 31, 67]. T1D is defined as an autoimmune disease characterized by a loss of insulin-producing β-cells, which exist in clusters known as islets of Langerhans in the pancreas. The progressive loss of β-cells reduces insulin release and eventually eliminates glycemic control [67]. Treatments have evolved from daily insulin injections, finger pricks and diet management to semi-autonomous, closed-loop systems integrating glucose monitors and insulin pumps. Collectively, these devices are referred to as an artificial pancreas (AP) [8]. Rather than targeting the β-cells themselves, AP technologies replace their critical functions artificially.

Pre-clinical studies show promising restoration of glucose responses using β-cell clusters generated from stem cells [79, 99]. However, endogenous β-cells in the pancreas receive parasympathetic innervation. Transplanted, stem cell-derived β-cell clusters lack this neural input. In this review, we will show the progress from pharmaceutical to bioelectronics to manage metabolic functions and further suggest a future direction towards biological neuromodulation using rationally-designed, multicellular biological circuits (*biocircuits* for short) of an autologous origin [85]. We will explore emerging biological engineering strategies to produce functional living tissue implants [53, 96] to restore or replace functional circuits lost due to injury or disease. Finally, we will propose a biocircuit strategy for the treatment of T1D, which integrates β-cell replacement therapy with advanced regenerative medicine to reinnervate the implanted tissue for better restoration of glycemic control.

## Neural control of metabolic function

**Fig. 2:**
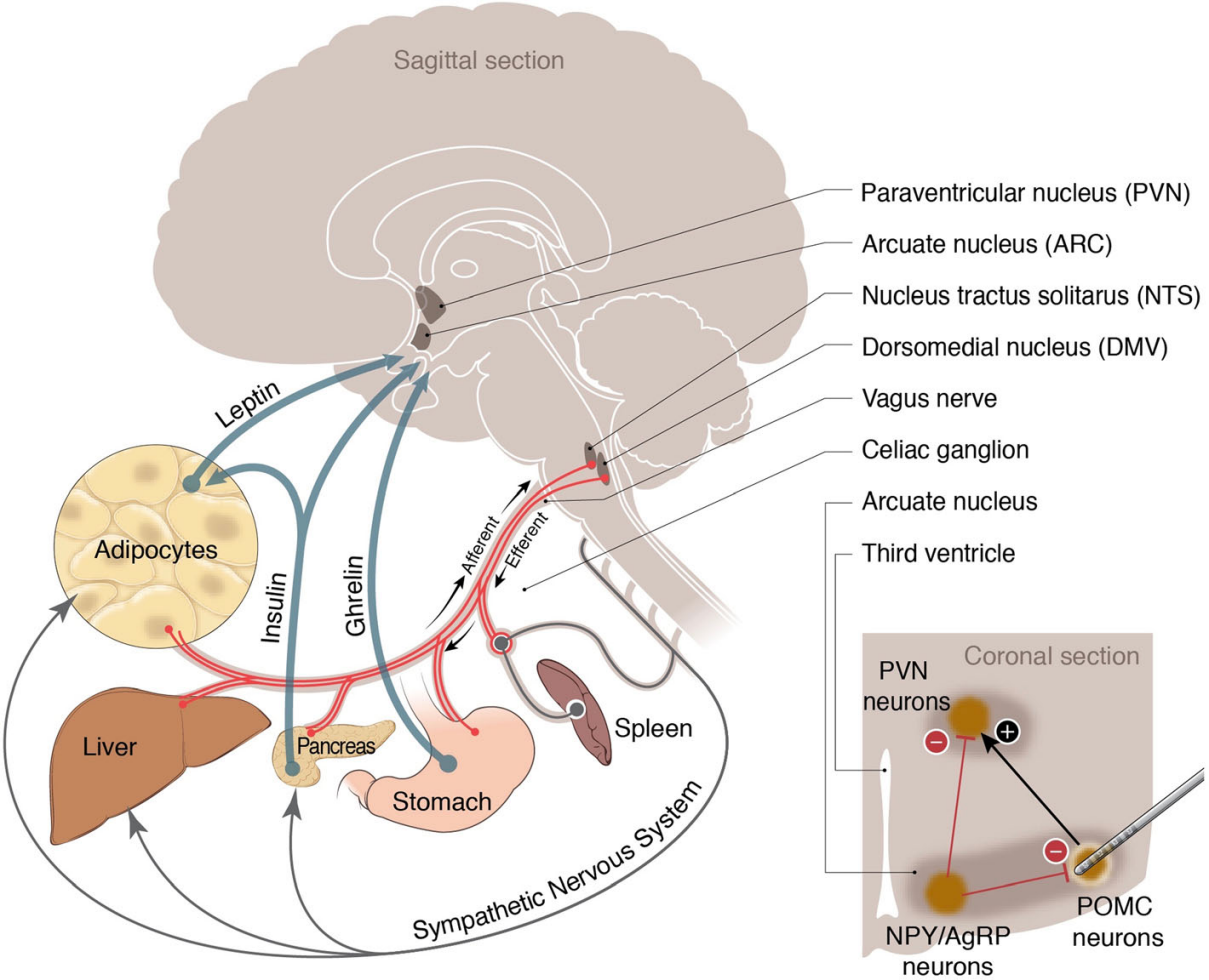
Neuroendocrine and neurometabolic circuitry regulation of metabolic functions. Both afferent and efferent pathways regulate energy balance through hormones and direct neural circuits. Ghrelin, insulin and leptin are the primary hormones that mediate the sensation of satiety and hunger by activating various populations of neurons in different regions of the brain. Autonomic innervations of metabolic organs are also depicted. SNS efferent fibers control hepatic and adipocyte metabolic pathways. Vagal afferents and efferent continuously monitor and regulate systemic metabolism. Cellular metabolism, including the production and release of cytokines from the spleen, responds to the sympathetic and parasympathetic convergences in the celiac ganglion. Inset, the NPY/AgRP and POMC neurons in the ARC of the hypothalamus inversely respond to these hormones and modulate the activation of the PVN neurons that in turn regulate feeding behavior and metabolic functions. Deep brain stimulation of POMC neurons ameliorates symptoms of diabetes in rat models, and therefore may provide a therapeutic avenue for neuromodulatory treatment of metabolic diseases. Image courtesy of Anthony S. Baker and Courtney Fleming, The Ohio State University^©^ 2019; produced with permission.

Regulating metabolism is a vital function for survival and requires the coordinated activities of many physiological systems. The central nervous system (CNS) is integral for the regulation of metabolism by directly sensing metabolic states and releasing neuroendocrine signals. The CNS also communicates with the body via cranial and spinal nerves through both efferent and afferent fibers. Both sympathetic and parasympathetic circuits influence metabolic functions, such as energy expenditure [42] and circulating levels of glucose in the blood [21]. In the following section, we will discuss the underlying circuitry by which the central and autonomic nervous systems (ANS) regulate metabolic functions (Fig. 2).

### CNS: hypothalamic control of metabolic activities

The brain constantly monitors the metabolic states of the body. Information from peripheral metabolic organs such as the pancreas, skeletal muscles and liver (Fig. 1a) is carried by visceral nerve fibers into the brain stem and subsequently relayed to the hypothalamus [87]. Circulating metabolites and hormones are also sensed directly by the hypothalamus [29], which responds to maintain metabolic homeostasis by neuroendocrine signaling [29, 87].

#### Hypothalamic circuits and neuronal populations

Different populations of neurons respond to metabolic cues to promote behavioral responses. Two important populations are the pro-opiomelanocortin (POMC) neurons and the agouti-related peptide/neuropeptide Y (AgRP/NPY) neurons [20]. POMC neurons in the arcuate nucleus (ARC) increase energy expenditure and reduce feeding behavior when responding to an internal energy state. AgRP/NPY neurons have the opposite effect of the POMC neurons in response to the same internal cues. The AgRP/NPY population do this by inhibiting POMC mRNA expression [77]. Activated POMC neurons result in a feeling of fullness and stop the behavior of eating, while activated AgRP/NPY neurons result in a feeling of hunger by the release of various hormones, including ghrelin and perhaps insulin [98]. POMC neuron activation depends on insulin concentration. Phosphate tyrosine phosphatase activity balances the amount of excitation and inhibition in these two populations [38]. AgRP/NPY and POMC are the main first order neurons that respond to leptin. Both insulin and leptin regulate metabolic functions, such as communicating energy states with the brain, suppressing appetite after eating and stabilizing blood glucose levels. Activation of the leptin receptor inhibits AgRP/NPY neurons, increases energy expenditure and maintains glucose homeostasis [45, 110]. Both insulin and leptin act as feedback signals to regulate food intake and maintain metabolic homeostasis through their inverse actions on AgRP/NPY and POMC neurons (Fig. 2).

The ARC in the hypothalamus contains both neuronal populations and has projections to the periventricular nucleus (PVN). Secondary neurons in the PVN play an important role in controlling the release of neuroendocrine signals to regulate blood glucose levels [64]. This network senses circulating hormones and regulates metabolisms [52]. Stimulation of these circuits allows for exogenous control of weight gain [74] and glucose metabolism [5]. Selective modulation of these distinct neuronal populations provides access to regain control of systemic metabolic functions.

### ANS regulation of metabolic functions

#### Visceral and cranial nerves

Neurometabolic circuitry between the hypothalamus and brainstem relay information about the states of the body through multiple pathways [68, 97]. Sensory information arrives in the nucleus tractus solitarius (NTS) from the periphery through the vagus nerve (see Fig. 1a). The afferent fibers of the vagus nerve can sense metabolites in the blood and various organs to convey the information to the CNS [34, 73]. Within the brain stem, reflex circuits respond to metabolic cues independently of the hypothalamus [15, 97]. Efferent fibers of the vagus nerve exit the CNS from the dorsal motor nucleus (DMN) of the vagus nerve and innervate every organ system in the body, including the brown adipose tissue (BAT) [93], liver [37] and pancreas [102]. Both the afferent and efferent fibers have the capacity to control metabolic functions. The carotid sinus branch of the glossopharyngeal nerve [95] has been implicated in neurometabolic reflexes. Cranial nerves can be accessed through less invasive means than deep brain regions and may provide more direct control over downstream metabolic targets. Therefore, they are attractive targets for neuromodulation to control metabolic functions [76].

Neuroimmune circuitry regulates the metabolic states of immune cells [25]. Both sympathetic and parasympathetic nerve fibers innervate metabolic and immune organs and tissues, including the splenic nerve terminals in the spleen (Fig. 1a), and may contribute to the pathophysiology of chronic inflammatory diseases. These neuroimmune circuits present an opportunity to resolve inflammation through targeted neuromodulation. Understanding the communications underlying neural controls of both inflammation and systemic metabolisms requires functional mapping of the ANS circuitry.

#### Sympathetic nervous system

The sympathetic nervous system (SNS) regulates energy expenditure, metabolite release and glucose homeostasis through noradrenergic signaling in the peripheral tissues and organs (Fig. 2). β-adrenergic receptors have been identified on numerous metabolic tissues and organs in the body, including the brown adipose tissue (BAT) [75], liver [26] and pancreas [7]. Sympathetic hyperactivation is commonly seen in obesity and diabetes [103]. SNS dysfunction may contribute to the pathophysiology of these diseases, and SNS activation can regulate glucose levels in the blood [21]. Neuromodulation to control SNS function is a potential intervention to prevent the progression of metabolic diseases.

#### Parasympathetic nervous system

Parasympathetic fibers innervate metabolic regulatory organs, such as the pancreas (Fig. 2). These neurometabolic circuits provide an exciting opportunity to intervene and control metabolic dysfunctions. Parasympathetic activity regulates β-cell insulin release in response to glucose. Vagus nerve terminals in the pancreas (Fig. 2) innervate β-cells in islets and release acetylcholine (ACh) which potentiates β-cell excitability [68, 102]. ACh alone does not cause the release of insulin, rather, activation of vagal nerve fibers makes the self-regulated system of insulin release by β-cells more effective in response to glucose.

#### Sensory axon reflexes

Sensory neurons innervating barrier surfaces [63, 106] dynamically regulate the metabolic states of immune cells. Bacteria activate sensory fibers directly in the skin during acute infection and decrease immune cell recruitment to the site and nearby draining lymph nodes [27]. Activation of these same type of sensory fibers regulates skin inflammation in psoriasis [86]. Selectively silencing sensory fibers in the lungs [100] alleviates allergic airway inflammation. While innate immune responses take on the order of minutes to hours (and adaptive immune responses take days to weeks), neural-immune reflexes can act on the order of seconds to allow for critical responses to immediate insults and pathogens. Controlling sensory nerves through this “axon reflex” [84] could allow for new, fast-acting anti-inflammatory bioelectronic interventions.

#### The cholinergic anti-inflammatory pathway

Autonomic regulation of systemic immunity began to be appreciated with the identification and isolation of ACh in the spleen [32] and demonstration that electrical stimulation of the splenic nerve increased ACh levels in the spleen [16]. Anatomical evidence reveals that structural contacts exist between sympathetic nerve terminals and immune cells in the spleen [44, 69], reviewed in [81]. These intimate connections between neurons and immune cells have been called the “neuro-immune synapses” [40, 41, 104].

ACh in the spleen reduces splenic inflammation leading to the notion of the “cholinergic anti-inflammatory pathway” [90, 91], reviewed in [105]. Splenic nerve terminals innervating the spleen (Fig. 2) release norepinephrine [69]. Specialized T-cells relay these incoming neural signals and release ACh to reduce macrophage activation [90]. Chronic systemic inflammation is among the leading risk factors for cardiovascular diseases (CVDs), which kill more than 2,200 people per day [12]. Reducing systemic inflammation has been shown to improve patient outcomes in CVDs [107]. Stimulating neural circuits to ameliorate splenic inflammation may provide a novel therapeutic avenue for patients.

## Pharmaceutical modulation of metabolic functions

Amphetamines demonstrate that pharmacological control of neurometabolic circuitry can be used to control metabolic functions. Many pharmaceutical interventions targeting neuronal activities alter metabolism based on the mechanism of action of amphetamines. Phentermine, marketed under the generic name ADIPEX-P^®^, is a sympathomimetic amine approved for the treatment of obesity [60] and triggers the release of norepinephrine and, to a lesser extent, dopamine and serotonin to increase energy expenditure and suppress appetite. This falls into a class of drugs called anorectics. However, neuromodulatory pharmaceutical treatments to control metabolic functions have many and often debilitating side-effects, including insomnia, pulmonary hypertension, and heart diseases [54]. Beyond weight-loss, controlling neurometabolic circuitry using pharmaceutical interventions is limited. Rather, treatments focus on restoring or replacing the functions lost due to the pathology of the disease, for instance, insulin replacement therapies for the treatment of diabetes. As with all pharmaceutical-based therapeutics, such hormone replacement therapies also have off-target effects. Additionally, many chronic diseases are or become resistant to pharmacological treatment. These challenges have led to advancements in the delivery systems used to reduce side-effects and drug resistance by delivering the drugs as needed. To highlight the significance of these advances, we will review the progress in the pharmaceutical management of T1D to demonstrate the capabilities and limitations of advanced pharmaceutical treatments.

### Pharmaceutical treatment of T1D

The discovery and isolation of insulin almost 100 years ago revolutionized the treatment of T1D and allowed patients to maintain a more stable glycemic index. Daily injections of long acting insulin represent the beginning of pharmaceutical treatment for T1D (Fig. 1b). For nearly 80 years, standard pharmaceutical-based therapy has been used to treat patients with T1D. Patients were still required to carefully maintain restricted diets and constantly measure their blood glucose levels, known as self-monitoring of blood glucose (SMBG). Advanced drug delivery systems, including glucose sensors and microneedle insulin pumps, revolutionized the management of T1D (Fig. 1b). Continuous glucose monitoring (CGM) and hybrid closed-loop systems allow patients to reduce their dietary restrictions and maintain more flexible lifestyles.

### Advances in drug delivery systems for the treatment of T1D

Advances in biosensors, microfabrication and closed-loop systems have dramatically improved the ability for patients with T1D to maintain blood glucose levels in healthy ranges. AP technology continues to improve by integrating CGM with microneedle insulin pumps to develop closed-loop hybrid systems [59]. However, SMBG is still required to calibrate interstitial glucose sensors for proper device function [67]. Prior to eating a meal, users must manually apply a bolus of insulin to prevent glucose spikes [35, 67]. Despite these remaining limitations, advanced drug delivery systems, including APs, have become the standard care for T1D and have greatly improved patient outcomes [46, 59].

Hybrid closed-loop systems for semi-autonomous glycemic control represent the state of the art in AP technology (Fig. 1b), which is currently the best available treatment for patients with T1D [46, 59]. CGM technologies have paved the way for such closed-loop systems [23]. The sensor measures the amount of glucose in the interstitial space in the skin, which correlates with blood glucose levels. While once patients had to perform SMBG eight or more times per day, current technology has reduced this down to two or fewer for calibrations. Hybrid closed-loop insulin delivery systems semi-automate the measurement and injection of insulin by integrating sensors, transmitters, insulin pumps, and devices to readout and control the system [33, 101].

Advanced pharmaceutical delivery systems have tremendous potential to help in the case of chronic administration of medication, however, many diseases or subgroups of patients become resistant to pharmacological interventions regardless of the delivery methods. Despite lower doses in targeted delivery systems, side-effects cannot be eliminated completely. In the case of immunosuppression therapies for example, the primary effect of the treatment can lead to infection and death. Collectively, these challenges have encouraged the development of innovative new therapeutic strategies. Increased appreciation for the role of the nervous system in the pathophysiology of numerous chronic conditions, including inflammation, autoimmune diseases and chronic pain, has led to the emergence of a new generation of medicine referred to as bioelectronic medicine or electroceuticals [43]. Rather than pharmacological modulation of diseases, bioelectronic medicine uses electrical control of the nervous system to ameliorate symptoms by targeting the dysfunctional neural activity responsible for exacerbating the disease pathology.

## Bioelectronic medicine – targeting the nervous system to control metabolic functions

Descending regulation of metabolism from the CNS is critical to maintain homeostasis throughout the body. Using deep brain stimulation (DBS, Fig. 1c) to control metabolic function could be used to control appetite, energy expenditure, and glycemic index through neuromodulation of the neurometabolic circuitry. Biointegrated electronic implants such as DBS devices could be used, for example, to target POMC neurons in the ARC (Fig. 2 inset). Additionally, case studies of Parkinson’s patients with DBS implants have shown a basal ganglia contribution to metabolic functions [55]. CNS-based neuromodulation using DBS provides an access point for bioelectronic therapeutics targeting metabolism.

Electrical stimulation of the vagus nerve (Fig. 1c) may restore glycemic control [2, 58, 76] and decrease hyperactive immune functions in chronic inflammatory diseases [61, 109], reviewed in [57]. Neurometabolic circuits allow for the targeted restorations of dysfunctional metabolic activities, including hyperglycemia and inflammation [58]. Neuronal control of systemic metabolism—including neuroendocrine release of hormones, central and peripheral nerve activations, and paracrine modulation of tissue and organ functions—provides multiple points of access for bioelectronic interventions to treat metabolic diseases (recently reviewed in [24]. Targeting neuroimmune circuitry can regulate the activation of immune responses through control of the neural-immune communications and cytokine signalings [25].

### CNS

Electrical stimulation of both the nucleus ambiguus and the DMN increases circulating levels of insulin [15, 56]. With the development of powerful new tools to modulate neural activities, we can functionally dissect the circuitry underlying neurometabolic regulations. Studies in rodents utilize optogenetic, chemogenetic and magnogenetic stimulation paradigms to selectively activate and inactivate specific neuronal populations [36]. Once unraveled, these convoluted networks may be targeted in patients for neuromodulation to control the associated metabolic functions.

DBS of the ARC (Fig. 2), which regulates appetite and energy expenditure, can ameliorate symptoms of diabetes in rodent models [74]. Electrical stimulation of glucose sensing neurons in the CNS [5] can control systemic glucose levels. Striatal dopamine also can regulate systemic glucose metabolism; and DBS in patients with diabetes results in increased insulin production and enhanced glycemic control following stimulation of the basal ganglia [55]. Percutaneous electrical neurostimulation of the T7 vertebrae [92] reduces blood glucose concentration, suggesting spinal control of systemic metabolic functions. Taken together, these studies reveal how neuronal regulations of metabolic functions can be used for bioelectronic interventions. Better understanding of the dysregulation in these circuits will improve our ability to effectively restore the associated neurometabolic functions [39].

### PNS

The vagus nerve innervates nearly every organ and tissue in the body and is a hub for autonomic regulation [25]. Vagus nerve stimulation (VNS, Fig. 1c) could likely reduce the global burden of diseases [47], primarily by ameliorating the symptoms of cardiovascular diseases [5]. Additionally, vagal efferent fibers innervate the pancreas to control the excitability of β-cells, thereby facilitating their release of insulin [2, 72]. ACh released by vagal nerve terminals activates β-cells through muscarinic ACh receptors in the presence of glucose [88]. Abdominal VNS restores glucose metabolism in diet-induced obesity [72]. ANS function plays an important role in the pathophysiology of obesity [49], through both vagal and SNS activities [103]. Further, autonomic neuropathy may exacerbate symptoms of diabetes [19]. Reflex circuitry, including the vagus and carotid sinus nerves, help to maintain metabolic homeostasis. Activation of these reflexes improves outcomes in diabetic rats [95]. Ultrasonic stimulation has also been used to elicit focused neuromodulation of peripheral nerves [30]. Vagus nerve stimulation can also have side effects, including infection, cough, hoarseness, voice alteration, and paresthesias [13]. However, these result primarily because of the implantation in the neck. More targeted stimulation of proximal and distal branches of the vagus nerve near the organ targeted could dramatically reduce these side effects. We expect bioelectronic medicine will continue to mature as a targeted and highly efficacious therapeutic intervention for metabolic diseases.

New tools for stimulating nerves are constantly being developed in the lab and tested in the clinic. Bioelectronic medicine has gained international attention in the past decade [43, 78]. Chronic activation of C-fibers may exacerbate disease pathology in rheumatoid arthritis through the antidromic release of pro-inflammatory neuropeptides [22, 65, 66]. Electrical stimulation of dorsal root ganglia in rats with collagen-induced arthritis significantly reduced swelling in the hind paw ipsilateral to the dorsal root that was stimulated [83]. Mesenteric ganglion stimulation alleviates intestinal inflammation in dextran sodium sulfate-induced experimental colitis via sympathetic innervation [108]. Electrical stimulation of the saphenous nerve below the knee [62] can either increase or decrease leukocyte rolling in the knee depending on the stimulation frequency. Additionally, electrical stimulation of sensory or “afferent” fibers of the vagus nerve mediate local inflammation in experimental arthritis via a multi-synaptic, CNS-sympathetic reflex circuit [9]. Taken together, using sensory and sympathetic nerves to control local inflammation represents a novel approach for treating refractory inflammatory diseases.

Systemic inflammation is regulated largely by splenic immune function. Stimulating various cranial nerves, including the vagus [25, 82, 84], reviewed in [24] and carotid sinus nerves [94] reduce splenic inflammation. Vagus nerve stimulation has produced promising results in clinical trials for rheumatoid arthritis [61] and irritable bowel diseases [109] likely by reducing neurogenic splenic inflammation. The celiac ganglion and splenic nerve circuitry (Fig. 2) have been extensively mapped [11, 17, 69, 70]. Coupling local and systemic immune controls through these circuits could provide patients with synergistic therapies that leave host defense intact while eliminating the harmful effects of inflammation.

### Devices for electrical stimulation – Electroceutical delivery systems

Bioelectronic medicine is based on the use of electronic devices to stimulate the brain and nerves in patients to restore organ and system functions. Metabolic dysfunctions underlie numerous disease states, from T1D to chronic inflammatory conditions. Neurometabolic circuitry regulates these systems to promote health, and their dysregulation results in pathology. Therefore, bioelectronic solutions ameliorate symptoms by restoring proper neuronal activities. Electrical stimulation of the nervous system can be achieved primarily through two broad categories, either CNS or PNS stimulation. Representative devices and commercial systems to achieve CNS or nerve stimulation are shown in Fig. 1c. DBS allows for the targeted electrical stimulation or silencing of deep structures in the brain, which is necessary to modulate the CNS neurometabolic circuitry. Nerve stimulators, for example targeting the vagus nerve, are far less invasive especially if the nerve resides near the skin. In both cases, artificial electronic devices are implanted to control and record bioelectric signals in the body.

As we have discussed, these technologies allow for the treatment of refractory conditions and have already shown tremendous clinical potentials for complex and chronic diseases. However, many of the limitations of bioelectronic medicine arise from the artificial nature of the electronic implants themselves [51]. Foreign body responses cause the body to mount immune responses against the artificial devices, which impede functional electrical coupling and eventually lead to a complete failure as the scar encapsulation is established. Artificial stimulation paradigms can also reduce the efficacy of the biotic-abiotic interface through cellular adaptation and changes in physiology. Finally, long-term maintenance of the hardware is required for both DBS and VNS systems, as wires break, batteries need to be replaced, and electrodes degrade. Life-long invasive surgeries are required and cause an increased chance of infection and other complications associated with the procedures.

Significant efforts from interdisciplinary teams of engineers, biologists and physicians are working to overcome these challenges. Smaller, softer and biomimetic materials substantially reduce immune responses and prolong the operation of artificial implants. Decreasing electrical current by using more physiologically-relevant stimulation paradigms reduces tissue damage and deleterious compensatory responses. Combined with engineering of higher-fidelity devices, these solutions may overcome many of the obstacles facing the efficacy of long-term bioelectronic implants for neural stimulation. However, bioelectronic medicine relies on structural connectivity between nerves and tissues to restore organ functions. In the case of many progressive and chronic conditions, tissues and specific cells are lost over the course of disease. For example, the progressive loss of β-cells in patients with T1D decreases insulin production and reduces glycemic control. During the so-called “honeymoon phase” following diagnosis of T1D, patients maintain some responsiveness to glucose, which reduces their reliance on exogenous insulin. The remaining β-cells during this period will still respond to increased ACh, therefore VNS may provide an improved glycemic control. Over time, bioelectronic interventions will become less and less efficacious. In progressive degenerative diseases such as T1D, ultimately, cell replacement or advanced regenerative medicine is the only option to restore the endogenous control of the lost functions.

Stem cell-derived β-cell replacement therapies are extremely promising techniques to restore insulin production in diabetic mouse models [79, 99]. However, even mature β-cell clusters do not fully recapitulate endogenous pancreatic β-cell responsiveness to glucose. One reason for this may be the lack of innervation and cholinergic modulation of the β-cell activity. Biologically engineered implants could integrate cholinergic neurons with β-cell clusters to provide innervated tissue replacements that better restore the endogenous functions through neuronal potentiation and modulation of the replaced cells (Fig. 1d). The fundamental limitation of bioelectronic medicine caused by the loss of neural fibers or target cell populations can be overcome through advanced regenerative medicine combined with functional living tissue implants [53, 96] to form integrated biocircuits [85] and may provide life-long solutions for chronic diseases such as T1D.

## Future direction: transplantable smart biocircuit implants

Biocircuit-controlled, smart functional living tissue implants made of autologous materials hold the promise to overcome the primary challenge of chronically implanted electronic devices, namely they are free from foreign body responses and rejection [85]. Such smart biocircuit implants constructed using patient-derived induced pluripotent stem cells (iPSCs) contain self-presenting immune molecules and therefore will seamlessly integrate into the host and provide physiological stimulation, thereby surmounting the difficulties in present biotic-abiotic interfaces. Long-term maintenance of these biocircuits will also not be required, as long-lived cells in the body, such as neurons, typically last a lifetime. Furthermore, no battery is required, as the implant is nurtured by the ingrown microvasculature. These advantages make biocircuits the optimal solution for engineering future long-term, autonomously responsive smart medical implants. The challenges that remain are to use biologically-inspired designs and biological engineering to manufacture functional biocircuits to achieve relevant therapeutic functions. In the following section, we will outline a potential application, as an example, for biocircuits to restore lost tissues and functions for patients with T1D.

### Biocircuit concept to treat T1D

Neuromodulation to control metabolic functions may provide new therapeutic avenues for the treatment of numerous refractory diseases. Dysfunctional neurometabolic circuits are rarely addressed in the current standards of care. However, structural and functional mappings of these circuits are required to provide the proper foundations for achieving symptom relief through exogenous neuromodulation. T1D has begun to transition from standard pharmaceutical intervention (i.e. insulin injections) to advanced technologies for drug delivery and monitoring, including systems of sensors and networked insulin pumps. Bioelectronic medicine continues to progress in the treatment of many other diseases using various neuronal interfaces to control both CNS and PNS functions (Fig. 1c). In the case of stem cell-derived β-cell replacement strategies for T1D, the transition from bioelectronic to biocircuit is possible (Fig. 1c and d).

Innervated, stem cell-derived β-cell transplants may provide a robust and life-long symptom management by resupplying both the lost cells and their control neural circuit. Recent advances in the vascularization of biologically engineered transplants [99] have drastically improved the glucose sensitivity and subsequent insulin release. A recent protocol has been developed to drive maturation of differentiated β-cell islets in vitro [79]. However, generating physiologically-relevant insulin responses to changes in blood glucose remains elusive. Here, we propose a novel approach to overcome this challenge. Using biologically-inspired engineering, we hope to improve the efficacy of replacement cells or tissues by fabricating innervated β-cell biocircuits (concept shown in Fig. 1d) to recapitulate the in situ functionality with a better fidelity.

β-cells are electrochemically active cells [6, 18, 50, 88, 89] and depolarize and release insulin upon activation by glucose. Because neighboring β-cells are connected by gap junctions, depolarization spreads throughout the network and across the islets [14]. This process coordinates the release of insulin to achieve an effective regulation of glycolysis required to maintain glucose homeostasis. Electrical stimulation of pancreatic tissues induces the release of insulin [1]. β-cell activity is also regulated by direct neural innervation. Vagal efferent fibers innervate the pancreas and islets. ACh released by vagus nerve terminals increases the release of insulin upon stimulation by glucose [2, 68, 73]. Both direct electrical stimulation of β-cells and neuromodulation of the vagus nerve provide insights into β-cell function. Islets in the healthy pancreas do not operate in isolation, rather, they are densely innervated by vagus nerve fibers. The most effective β-cell replacement strategies involve the differentiation of mature β-cells [79], self-condensing of vascularized islets [99], and transplantation under the skin of the host. Although more effective and free from host rejection, these implants do not exhibit full glucose sensitivity. We hypothesize that the limited insulin response to glucose arises from the lack of innervation found in the healthy pancreas. Integrating biocircuits into β-cell replacement therapies (Fig. 1d) may thus restore the full glycemic control dynamics to patients with T1D.

Such biocircuit-augumented islet transplants may one day be used as a replacement therapy for T1D. Although still in the early stages of preclinical research, transplanted islets greatly improve the glycemic control in animal models of diabetes. However, they lack the important cholinergic innervation found in situ. Biocircuit-augumented islet transplants containing mature, vascularized and innervated β-cells will better mimic the endogenous glycemic control dynamics inside the pancreas. Such an attempt to restore the endogenous release of insulin could provide a lifelong relief for patients with T1D and may one day become the standard care for T1D.

## Conclusions

In this review, we have revealed the technological progression from pharmaceutical to bioelectronic medicine as targeted and precise therapeutics for refractory diseases characterized by dysregulation of metabolic functions. Despite the enormous progress in miniaturization and biomaterials, electronic medical implants still suffer the long-term challenges of host rejection, artificial stimulation, and deterioration. Therefore, we have proposed a succeeding solution of biologically engineered smart biocircuit implants. Furthermore, looking through the lens of history, we envision that this technological succession will lead to a future in which rationally designed, multicellular biocircuits will allow for the engineering of autonomously responsive medical implants to replace and restore functions to tissues lost in the pathology of metabolic diseases. Both T1D and chronic inflammatory diseases share similar characteristics in that metabolism, defined as cellular catabolic and/or anabolic processes, is disrupted, leading to systemic complications. Neurometabolic circuitry provides many access points for the neuromodulatory treatment of such diseases. Targeting neurometabolic circuitry using transplantable biocircuits holds a great promise to restore both the lost cells and functions, as well as providing life-long, seamlessly biointegrated prosthetics for the patients.

## Data Availability

N/A

## Abbreviations

ACh: Acetylcholine
AgRP/NPY: Agouti-related peptide/neuropeptide Y
ANS: Autonomic nervous system
AP: Artificial pancreas
ARC: Arcuate nucleus
BAT: Brown adipose tissue
CGM: Continuous glucose monitoring
CNS: Central nervous system
CVD: Cardiovascular diseases
DBS: Deep brain stimulation
DMN: Dorsal motor nucleus
iPSC: induced pluripotent stem cells
NTS: Nucleus tractus solitarius
POMC: Pro-opiomelanocortin
PVN: Periventricular nucleus
SMBG: Self-monitoring of blood glucose
SNS: Sympathetic nervous system
T1D: Type 1 diabetes
VNS: Vagus nerve stimulation
